# A study on commuters’ public transportation mode choice behavior in river valley-type cities considering terrain spatial perception: evidence from Lanzhou, China

**DOI:** 10.1038/s41598-024-64062-z

**Published:** 2024-06-09

**Authors:** Mengxing Fan, Jinping Qi, Xiangdong Zheng, Hongtai Shang, Jiayun Kan

**Affiliations:** 1 Mechatronics T&R Institute, Lanzhou Jiaotong University, Lanzhou, 730070 China; 2 Engineering Technology Center for Information of Logistics and Transport Equipment, Lanzhou, 730070 Gansu China; 3 Gansu Industry Technology Center of Logistics and Transport Equipment, Lanzhou, 730070 Gansu China; 4 Lanzhou Rail Transit Co., LTD., Lanzhou, 730030 China; 5 School of Electronic and Information Engineering, Lanzhou Jiaotong University, Lanzhou, 730070 China

**Keywords:** Psychology and behaviour, Human behaviour, Applied mathematics, Statistics, Software

## Abstract

Existing research rarely examines the subjective and objective built environment of river valley-type cities in relation to travel mode choice, particularly overlooking the heterogeneity among travelers in these cities. In this paper, based on questionnaire survey data and built environment data, terrain spatial perception (TSP) is introduced to expand the theory of planned behavior (TPB), and a Structural Equation Model (SEM) is established. Factor analysis and path analysis are conducted using SPSS and AMOS to estimate latent variables. An integrated model of SEM and random parameter Logit model (RPLM), which can not only analyze the psychological perception factors of commuters in river valley-type cities but also consider the heterogeneity of psychological perception, was constructed to analyze the impact of personal attributes, objective built environment factors, and psychological latent variables on the commuting mode choice behavior of public transport users in river valley-type cities. The results indicate that the five observation indicators corresponding to the proposed terrain spatial perception latent variables can better explain the terrain spatial perception of commuters in river valley-type cities. Different from plain cities, the subjective and objective built environment of river valley-type cities notably influence the travel behavior of commuters. Moreover, the parameters of terrain spatial perception follow a normal distribution, indicating that the sensitivity of different commuters to the terrain spatial perception of river valley-type cities is heterogeneous. The results of our study can provide a reference for alleviating traffic issues in valley cities.

## Introduction

Commuting travel, as the primary travel demand for passengers in urban public transportation, reflects the travel efficiency of public transportation. Commuting in China’s cities presents several disadvantages, including long distances, extended travel times, and imbalanced supply and demand of travel resources, particularly in cities located in rivers and valleys. Constrained by the narrow terrain and the division of the river in the region, the availability of road resources in river valley-type cities are limited compared to those in an ordinary city. This leads to widespread phenomena such as rapid growth in car development and inadequate development of a comprehensive public transport system. Merely expanding the road network by building urban roads cannot fundamentally solve the urban traffic problem in river valley-type cities.

The subway plays a crucial role in public transportation, helping to alleviate urban traffic issues. It offers the advantages of a large capacity, environmental friendliness, and no use of road resources. Additionally, the high speed of the subway enables it to reduce spatial distance and expand the activity space of residents in river valley-type cities^1^. The axial distance of the transportation corridor in a river valley-type city is significant and exhibits a clear agglomeration effect, which is also highly favorable for the development of a subway^2^. Based solely on the existing transportation facilities, promoting the subway as the primary mode of transportation in a river valley-type city can enhance urban transportation efficiency and alleviate traffic problems. Therefore, how to encourage commuters in river valley-type cities to use the subway for their daily commute?

In general, commuters will carefully consider various influencing factors and ultimately choose the optimal mode of travel^3^. Understanding the factors that influence commuters’ choice of public transportation in river valley-type cities can provide valuable insights for accurately formulating traffic policies and optimizing the public transportation environment, which can help promote the subway’s usage and alleviate the traffic problems in river valley-type cities. This paper addresses the following research question: What are the main factors that influence commuters’ choice of public transportation mode in river valley-type cities? To what extent do these factors influence? What measures might help alleviate traffic problems in river valley-type cities? We will address these questions in the following sections. In the part of "Literature review", we review the factors and methods discussed in the existing literature and identify the limitations of the current research. In the part of "Method", we elaborate on the theories and models used in the article. In the part of "Research area and data", we introduce the characteristics of the research object, the indicators, and variables selected to describe these characteristics, and provide a description of the data collected through the questionnaire survey. In the part of "Results" and "Discussion", we solve the model and analyze the results.

## Literature review

### Influencing factors of travel mode choice

The factors that affect the choice of commuting mode have always been a focal point for optimizing urban traffic structures. Commuters’ travel intention will vary based on individual characteristics, objective built environment features, and psychological perception. In terms of individual socioeconomic attributes, the residence time of commuters in mountainous cities with unique terrain is also considered to have a significant impact on travel choice^4^. In addition, scholars also believe that modifying the built environment can change travel choice behavior of individual^5^. Yun et al.’s study demonstrated that the road slope in mountain cities and whether the travel time is during peak periods significantly influence the choice of travel mode^4^. The narrow junctions of urban core groups and the obstacles of rivers in river valley-type cities have been proven to have an impact on travel^6^. The object characteristic variable varying with the scheme is also thought to affect commuters’ mode choice^7^.Studies have shown that, in addition to the objective built environment factors, travelers’ subjective perception of the built environment also significantly impacts travel behavior^8,9^. In terms of psychological perception factors, Fu et al. examined the significant impact of travelers’ subjective norms (SN), behavioral attitudes (BA) and perceived behavioral control (PBC) of travel modes decisions^10^. Mou et al. believes that SN will encourage car travelers to shift to public transportation^11^. Narrowing the gap between public transport and private cars in the level of travel service and improving travelers’ BA towards public transport are effective measures to encourage commuters to choose public transport^12,13^. Hu et al. holds the opinion that PBC, such as the degree of familiarity with public transport routes and the degree of convenience and freedom in choosing public transport have a significant impact on public transport travel intention^14^. It is worth mentioning that Xiong et al. proposed terrain perception for road slope in mountainous cities, confirming its significant impact on travel mode choice^15^.

### Analysis method of travel mode choice

Travel mode selection and choice forecasting are crucial components of traffic planning. It’s a complex process in which different theories and methods are used to analysis. Both domestic and foreign scholars primarily utilize discrete choice models, particularly the Logit model and its variations, as well as the Structural Equation Model (SEM). The discrete choice model is based on individual travel data modeling, which provides predictions that are closer to the objective reality. Pan et al. and Vedagiri both developed the binary Logit model for private car and bus travel and plotted the travel mode shift probability curve based on changes in parking charges and service levels of the two modes, respectively^16,17^. Zhang et al. compared the estimation results of the random parameter Logit model (RPLM) and Nested Logit models and found that RPLM can more accurately reflect the heterogeneity of travel cost sensitivity of different Commuters^18^. However, the traditional discrete choice model cannot explain the potential variables of psychological perception such as attitude and perception of Commuters. Therefore, the influence of psychological latent variables such as traveler’s psychological attitude and perception and preference for safety, convenience and flexibility on travel mode choice is quantified by establishing the structural equation model^19–21^. However, using only a single SEM analysis may lead to overlooking the correlations and interrelationships between latent variables, as well as between latent variables and other attribute variables. This oversight can result in misinterpreting potential categories and repeating factors unnecessarily^22,23^.To more accurately describe unobservable factors, scholars have integrated the Logit model with the SEM based on the theory of planned behavior (TPB) was developed. It was determined that commuters’ attitudes, cognition, norms, and service level of travel mode significantly affect individual travel mode choice intention and behavior^24,25^.

In summary, there is a wealth of literature on travelers’ choice of travel mode, but few studies consider travelers’ perception of the built environment, particularly those that do not consider the unique terrain perception in valley-type cities. At the same time, scholars only use either a single structural equation model or a logit model, and rarely combine the advantages of both. In addition, the general logit model cannot avoid the constraint of Independence of Irrelevant Alternatives (IIA) or account for the heterogeneity of travel individuals, which is inconsistent with the actual travel situation. The travel space of commuters in river valley-type cities differ from those in plain cities, and the study on the travel mode selection of the river valley-type city commuters cannot simply rely on findings from other cities. Therefore, this study introduced the terrain spatial perception (TSP) to extend TPB to account for the unique characteristics of river valley-type cities. Based on this, SEM was used to analyze the potential psychological factors influencing travel intention. The psychological latent variables obtained from the SEM were integrated into the RPLM along with individual attributes and built environment attributes. An integrated SEM-RPLM was established to analyze travel intentions and behaviors, encompassing both latent and explicit variables, and taking into account the heterogeneity of mental perceptions among commuters in river valley-type cities.

## Method

The methods in this section were developed following applicable global standards’ guidelines and rules.

### Theory of planned behavior

TPB is commonly used to study commuters’ mode choice behavior in traffic-related research^26,27^. In this study, behavior and intention may be influenced by these factors. First, SN refers to a commuter’s perception of how their travel behavior may change in response to support or pressure from relatives, friends, and society. Second, BA includes the commuter’s evaluation of the public transportation mode. Third, PBC refers to the perceived difficulty or ease for an individual to choose a specific mode of public transportation. However, studies have shown that in addition to the aforementioned factors, other psychological factors can also affect the behavior intention (BI)^28^, such as terrain perception^15^. Therefore, based on the characteristics of river valley-type cities, we incorporated the TSP to expand TPB, and proposed the following hypotheses, H1 ~ H4: SN, BA, PBC, and TSP will significant influence travel mode choice BI.

### Structural equation model

SEM is a crucial method for multivariate analysis and is conducted using AMOS software. Compared with traditional regression analysis, SEM has the advantage of simultaneously estimating the relationships among multiple variables in a single model simultaneously and testing multiple hypotheses within a single structural model. SEM mainly consists of two parts: the measurement model and the structural model.

The measurement model describes the latent variables through the corresponding observed variables.

1 $$x = \Lambda_{x} \xi + \varepsilon$$

2 $$y = \Lambda_{y} \eta + \varepsilon$$

where $x$ and $y$ are the vectors of observed variables of exogenous latent variables and endogenous latent variables, respectively; $\Lambda_{x}$ is the factor loading matrix of $x$ to $\xi$; $\Lambda_{y}$ is the factor loading matrix of $y$ to $\eta$; $\xi$ and $\eta$ are the vector composed of exogenous latent variables endogenous latent variables, respectively; $\varepsilon$ is vector of observation error.

The structural model utilizes exogenous latent variables to elucidate endogenous latent variables.

3 $$\eta = {\rm B}\eta + \Gamma \xi + \zeta$$

where ${\rm B}$ is the structural coefficient matrix of endogenous latent variables; $\zeta$ is exogenous the structure coefficient matrix of latent variable ; ζ is the residual vector of the model.

In order to fully consider the information contained in each observed variable in the latent variable, the weight of the loading coefficient between the latent variable and its observed variable is normalized^29^. Taking a latent variable $\xi$ representing selection behavior as an example, this latent variable $\xi$ corresponds to n observed variables, $X_{1} ,X_{2} , \cdots ,X_{n}$. The characteristic expression of the latent variable concerning its internal observed variables is obtained as shown in the formula.

4 $$\left\{ \begin{gathered} {\rm A}_{{X_{1} }} = \frac{{\Lambda_{{X_{1} }} }}{{\Lambda_{{X_{1} }} + \Lambda_{{X_{2} }} + \cdots + \Lambda_{{X_{n} }} }} \hfill \\ {\rm A}_{{X_{2} }} = \frac{{\Lambda_{{X_{2} }} }}{{\Lambda_{{X_{1} }} + \Lambda_{{X_{2} }} + \cdots + \Lambda_{{X_{n} }} }} \hfill \\ \vdots \hfill \\ {\rm A}_{{X_{n} }} = \frac{{\Lambda_{{X_{n} }} }}{{\Lambda_{{X_{1} }} + \Lambda_{{X_{2} }} + \cdots + \Lambda_{{X_{n} }} }} \hfill \\ \end{gathered} \right\}$$

where $X_{n}$ is between the observed variable $X_{n}$ and its latent variable $\xi$.

Then, the observed values of each observed variable are substituted into Eq. (5).

5 $$\xi = {\rm A}_{{X_{1} }} X_{1} + {\rm A}_{{X_{2} }} X_{2} + \cdots + {\rm A}_{{X_{n} }} X_{n}$$

The adaptation values of latent variables can be obtained. After solving the latent variable adaptation value of the SEM model, it is necessary to substitute it into the RPLM for integration.

### Random parameter logit model

For the analysis of travel mode choice behavior, scholars commonly utilize the logit model. However, the explanatory variables in the logit model only include the subject characteristic attribute variables that do not change with the selected scheme, which is inconsistent with the actual situation. The explanatory variables of the RPLM consist of both the subject characteristic attribute variables and the object characteristic attribute variables that vary according to the scheme^30^. In addition, the parameters of its utility function are randomly determined and subject to specific distribution laws. As a result, the RPLM mitigate the bias caused by IIA^31^.

### Integration model of SEM-RPLM based on TPB

**Figure 1:**
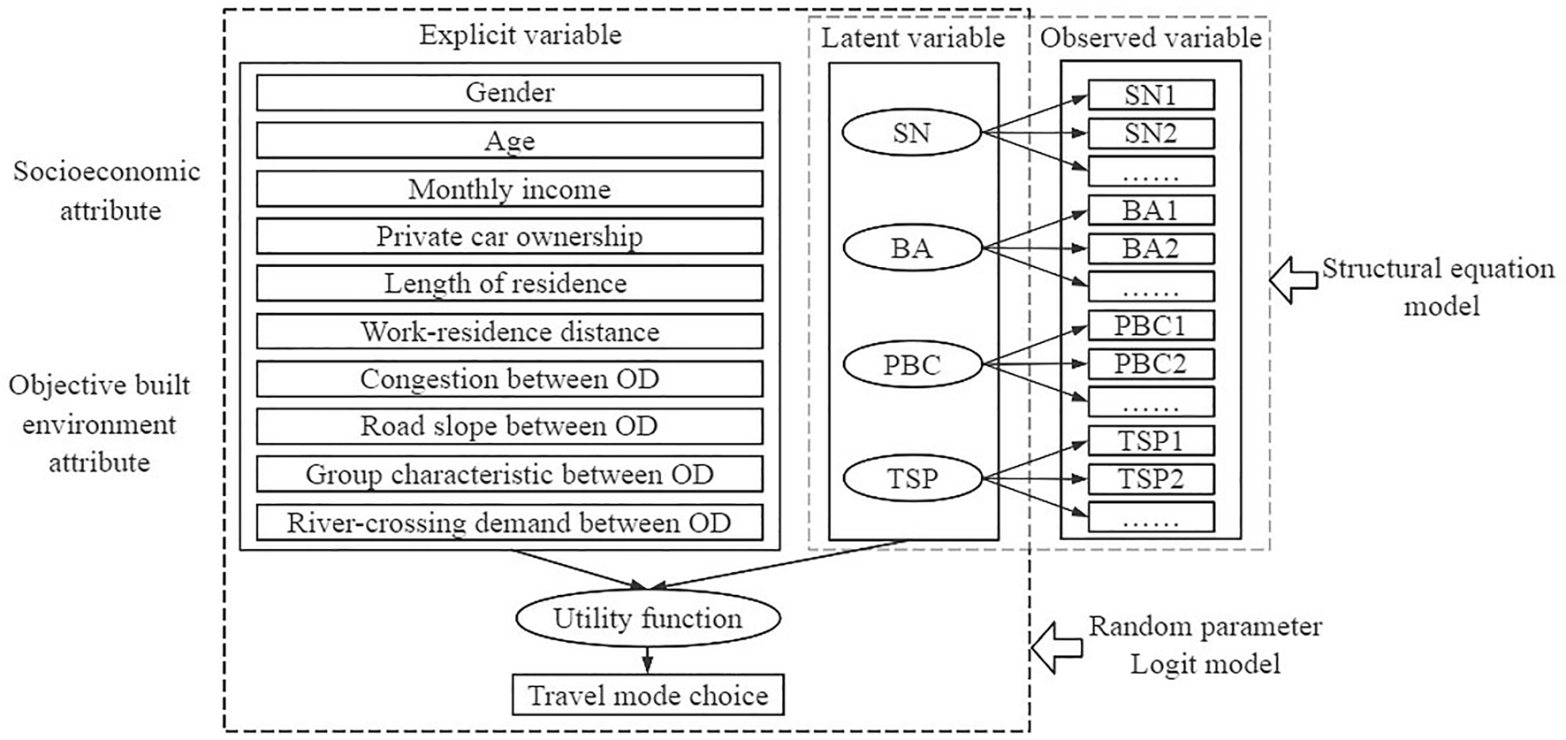
Theoretical model. Figure depicts the integration theoretical model of SEM-RPLM in this paper.

SEM can quantify the psychological potential factors of commuters. As a result, many researchers use SEM in the exploration of travel behavior^21^. In this paper, however, many factors are comprehensively considered. Therefore, solely utilizing the structural equation model to estimate the relationship between various factors simultaneously will overly complicate the established model relationship and require extensive calculations. Moreover, using only a single SEM analysis may result in misinterpreting potential categories and repeating factors unnecessarily as we have discussed in the part of :"Literature review"^23^. RPLM is well-suited for studying travel mode choice, but it does not accurately account for the influence of commuters’ perceptions, attitudes, and other psychological variables on travel behavior^32^. By comparing the existing literature, it can be predicted that the inclusion of psychological factors can more effectively explain individual mode choice behavior than solely considering individual factors and built environment factors^33,34^. For example, a younger commuter may perceive the slope of a road differently than an older commuter in river valley-type cities^15^. So it is necessary to include psychological perception factors. Therefore, this paper focuses on Lanzhou City as the research subject, based on the extended TPB, SEM is introduced to depict the impact of potential psychological perception variables on travel decision-making. SEM-RPLM analyzes the influence of commuters’ psychological attributes, travelers’ personal attributes, and objective built environment attributes on BI travel mode choice collectively. In addition, considering the heterogeneity among commuters in river valley-type cities also helps reduce the bias caused by IIA (see Fig. 1).

If we assume that the set of alternatives for a traveler $n$ is $A$ and the utility of scheme $i$ is $U_{in}$,$i \in A$, then the utility function for scheme $i$ can be expressed as Eq. (6). In the RPLM, $e_{in}$ is divided into two parts, one is a random term that allows each choice to have correlation and heterogeneity, and the other is a random error term.

6 $$U_{in} = X_{in} \beta_{n} + e_{in} = X_{in} \mu_{b} + (X_{in} \eta_{b} + \varepsilon_{in} )$$

where $X_{in}$ denotes the measurable part of the choice’s utility, $\beta_{n}$ and $e_{in}$ are random influence variables that cannot be directly measured. $\mu_{b}$ is the mean of $\beta_{n}$, $\eta_{b}$ is the random term of $\beta_{n}$, and $\varepsilon_{in}$ is the random error term.

The maximum likelihood estimation method is used to estimate the parameters of RPLM. If the parameter is set to random, then the probability of the traveler $n$ choosing scheme $i$ can be expressed as Eq. (7).

7 $$P_{in} = \int {\frac{{\exp \left( {\beta_{n} X_{ni} } \right)}}{{\sum\limits_{i \in A} {\exp \left( {\beta_{n} X_{ni} } \right)} }}f(} \beta_{n} |\theta )d\beta_{n}$$

where $f(\beta_{n} |\theta )$ is the probability density function obeying a certain distribution. The common distribution forms of $f(\beta_{n} |\theta )$ are normal distribution, uniform distribution, triangular distribution, and logarithmic normal distribution. Among these, the normal distribution is a more commonly used form of distribution^7,29,35^.

### Ethical approval

The implement at this study followed the principles of the Helsinki Declaration and the study was approved by Ethics Committee of Lanzhou Jiaotong University in manuscript file.

## Research areas and data

### Research area

Lanzhou is the capital of Gansu Province and the second-largest city in northwest China. Situated at the crossroads of the northwest, the city has benefited from the Silk Road, making it an important transportation hub and tourist destination. The city’s terrain is elevated in the northwest, with the Yellow River flowing from the northwest to the southeast, traversing the entire area. This forms a bead-shaped river valley-type with alternating gorges and basins, making it a typical river valley-type city. In summary, this survey sample closely aligns with the actual population distribution and urban travel characteristics of Lanzhou^36^.

Compared with plain cities, river valley-type cities have a higher degree of separation between workplace and residence and a larger road slope^6,37^. The traffic development balance of river valley-type cities is poor^38^. The transportation network and facility development of each urban center are better than that of each urban edge, leading to differences in the convenience of traveling between urban groups and within group. The non-linear coefficient of river valley-type city’s road network is high, characterized by complexity, numerous broken roads, and one-way streets^39^. Moreover, residents are required to traverse the city center to facilitate communication between the two ends of the city, leading to unnecessary detours^40^. In the river valley-type city, the river runs through the city in a direction consistent with the main road^41^. As a result, road traffic travelers in the river valley-type city must take more detours to locate the bridge and cross the river compared to travelers in an ordinary plain city. The peak hours in river valley-type cities tend to last longer compared to plain cities of the same scale^6^. This is primarily attributed to the underdeveloped traffic infrastructure at the periphery of river valley-type cities, narrow junctions of urban groups, unidirectional passenger flow, extensive communication between the central area and other urban groups, and numerous detours caused by rivers and a complex network^40^.

Due to significant variations in traffic characteristics and background, the study of travel mode choice behavior in river valley-type cities cannot fully rely on the index system used for general cities of similar scale. Therefore, on the basis of the mature scale of the existing TPB^25,26^, five measurement indices for the terrain spatial perception of the river valley-type city are added^42^. The observed variable corresponding to the latent variable are described in Table 1.

**Table 1:** Description of observed variable. These question descriptions are aimed at the public transport mode that respondents use most during the survey month.

Latent variable	Description of the observed variable corresponding to the latent variable
Subjective norms	
SN1	My family and colleagues supported me in choosing this public transportation mode
SN2	The social policy supports my choice of choosing this public transportation mode
SN3	The mobility decisions of the people around me also affect my travel decisions
Behavior attitude	
BA1	It is safer to choose this travel mode in river valley-type cities
BA2	It is more convenient to choose this travel mode in river valley-type cities
BA3	It is more economical to choose this travel mode in river valley-type cities
BA4	It is more comfortable to choose this travel mode in river valley-type cities
BA5	It is more accessible to choose this travel mode in river valley-type cities
Perceived behavior control	
PBC1	The cost of choosing this public transportation mode can be afforded
PBC2	It is simple for commuters to choose this public transportation mode
PBC3	The choice of public transportation mode depends entirely on myself
Terrain spatial perception	
TSP1	The detour of this public transportation mode is relatively fewer in river valley-type cities
TSP2	This public transportation mode is more convenient to travel between cities groups
TSP3	This public transportation mode is less affected by the rivers of river valley-type cities
TSP4	The public transportation mode is less affected by the road slope in river valley-type cities
TSP5	The public transportation mode is less affected by the congestion in river valley-type cities
Behavior intention	
BI1	It is optimal for commuters in river valley-type cities to choose this public transportation mode
BI2	I am willing to often choose this public transportation mode in river valley-type cities
BI3	I have planned to take this public transportation mode as the preferred one

## Research data

The study focuses on commuters in Lanzhou, a typical river valley-type city in China. The research was conducted in the central streets of three urban clusters of Lanzhou from December 11, 2023, to December 15, 2023, using an offline questionnaire which consists of two parts.

The first part of the questionnaire evaluates SN, PBC, BA, TSP, and BI, using a Likert 5-point scale. The Revealed Preference (RP) survey method was used to analyze the behavioral choice of travel modes in the second part which including three subsets. Given the characteristics of river valley-type cities, including significant separation between workplace and residence^37^, steep road slope^6^, obvious urban grouping characteristic^41^, the necessity of finding bridges to cross the river^41^, and prolonged peak periods^41^, factors such as workplace-residence distance, road slope, travel grouping characteristic, river crossing demand, and congestion between origin and destination(OD) were considered. The socioeconomic characteristics of individual commuters include gender, age, monthly income, length of residence, and ownership of private car. The set of objective built environment attributes includes workplace-residence distance, road slope, travel grouping characteristic, river crossing demand, and congestion between OD of the most common commute. The built environment data obtains POI information through the Gaode map^7^. The travel options subset includes the following choices: subway, taxi, and bus. The respondents’ most commonly used monthly public transport commuting mode was selected as the result. Public transport refers to all modes of transportation that are available to the public and provide transportation services^43,44^. Compared with plain cities of the same size, valley-type cities exhibit a greater degree of separation between workplace and residential areas, with medium and long-distance commuting trips constituting the majority. Therefore, this study only focuses on medium and long-distance public transportation^39^. Questionnaires with travel mode choices outside the specified set will be excluded. In view of the fact that there is no significant difference between e-hailing cars and taxis in terms of safety, convenience, economy, and comfort, this paper only includes taxis as a representative of the two in the selection set.

**Figure 2:**
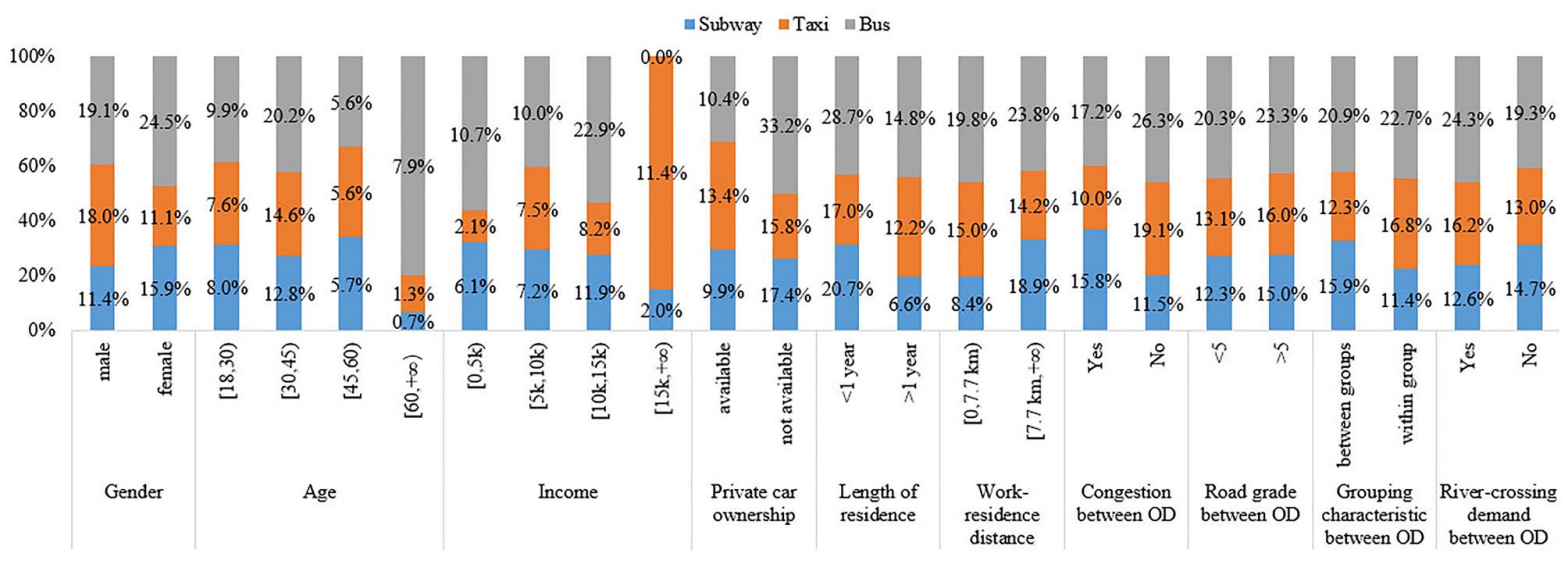
Statistical description of sample respondents.

All participants received comprehensive information about the study and willingly signed informed consent forms before participating in the research. A total of 2400 questionnaires were distributed. Invalid questionnaires containing missing values and extreme continuous values were excluded^45^. This resulted in 2244 valid questionnaires, achieving an effective response rate of 93.50%, which met the sample size requirement^46^. Descriptive statistics of sample are shown in Table 2. The sample characteristics are depicted in Fig. 2.

**Table 2:** Descriptive Statistics.

Variable	Definition	Mean	Std
Subject characteristic attribute variable (Variable that do not change with the scheme)			
Gender	1 male,0 female	0.485	0.500
Age	1 [18,30), 2 [30,45), 3 [45,60), 4 [60, + ∞)	2.112	0.899
Monthly income(CNY)	1 [0,5 k), 2 [5 k,10 k), 3 [10 k,15 k), 4 [15 k, + ∞)	2.507	0.947
Private car ownership	1 not available, 0 available,	0.337	0.473
Length of residence	1 length of residence > 1 year, 0 length of residence < 1 year	0.667	0.477
Workplace-residence distance/km	1 [0,7.7 km) , 0 [7.7 km, + ∞)	0.568	0.495
Road slope between OD/%	1 [0,3), 2 [3,6), 3 [6,9), 4 [9,12), 5 [12, + ∞)	2.723	1.240
Grouping characteristic between OD	1 within urban group, 0 between urban groups,	0.491	0.500
Congestion between OD	1 yes, 0 no	0.430	0.495
River-crossing demand between OD	1 yes, 0 no	0.469	0.499
Object characteristic attribute variable (Variable that vary with the scheme)			
Subway	1 very disagree, 2 disagree, 3 neutral, 4 agreed, 5 very agree		
Subjective norms		4.067	0.706
Behavior attitude		4.036	0.679
Perceived behavior control		4.178	0.601
Terrain spatial perception		3.603	0.783
Behavior intention		3.904	0.492
Taxi			
Subjective norms		3.857	0.771
Behavior attitude		3.799	0.853
Perceived behavior control		3.772	0.899
Terrain spatial perception		3.398	0.742
Behavior intention		3.657	0.652
Bus			
Subjective norms		4.036	0.725
Behavior attitude		4.027	0.716
Perceived behavior control		3.746	0.680
Terrain spatial perception		3.674	0.886
Behavior intention		3.639	0.503

In order to ensure the validity of the subsequent modeling research, it is necessary to test the reliability and validity of the scale data in the questionnaire by IBM SPSS software.

As indicated in Table 3, the questionnaire data successfully passed the Cronbach’s and Kaiser-Meyer-Olkintest (KMO) test^47^. This indicates that the data met the criteria for multivariate normality and sampling adequacy, demonstrating reliable data quality^48^. Moreover, the cumulative variance interpretation rates were greater than 40%^49^. Utilizing the composite reliability (CR) and average variance extracted (AVE) to assess the reliability and validity. The CR of each dimension exceeds 0.7, and the AVE is higher than 0.4, indicating that the measurement index of the theoretical framework proposed in Table 3 demonstrates good reliability and validity^50^.

**Table 3:** Reliability and validity tests.

Latent variable	Factor load coefficient	Cronbach’s Alpha	AVE	CR	KMO	Bartlett’s Test of Sphericity	Total variance explained cumulative
Subjective Norms							
SN1	0.548	0.765	0.533	0.772	0.903	0.000	72.292%
SN2	0.699						
SN3	0.822						
Behavior attitude							
BA1	0.553	0.821	0.511	0.835			
BA2	0.643						
BA3	0.848						
BA4	0.730						
BA5	0.807						
Perceived behavior control							
PBC1	0.797	0.782	0.566	0.794			
PBC2	0.604						
PBC3	0.706						
Terrain spatial perception							
TSP1	0.723	0.870	0.582	0.873			
TSP2	0.825						
TSP3	0.809						
TSP4	0.697						
TSP5	0.675						
Behavior intention							
BI1	0.769	0.853	0.679	0.864			
BI2	0.736						
BI3	0.744						

Data source: Cronbach’s Alpha, KMO, and Total variance explained cumulative obtained by the authors from the results of SPSS 26.0 software runs. AVE and CR obtained by the authors’ calculations based on formulae.

## Result

### Travel mode choice intention

**Figure 3:**
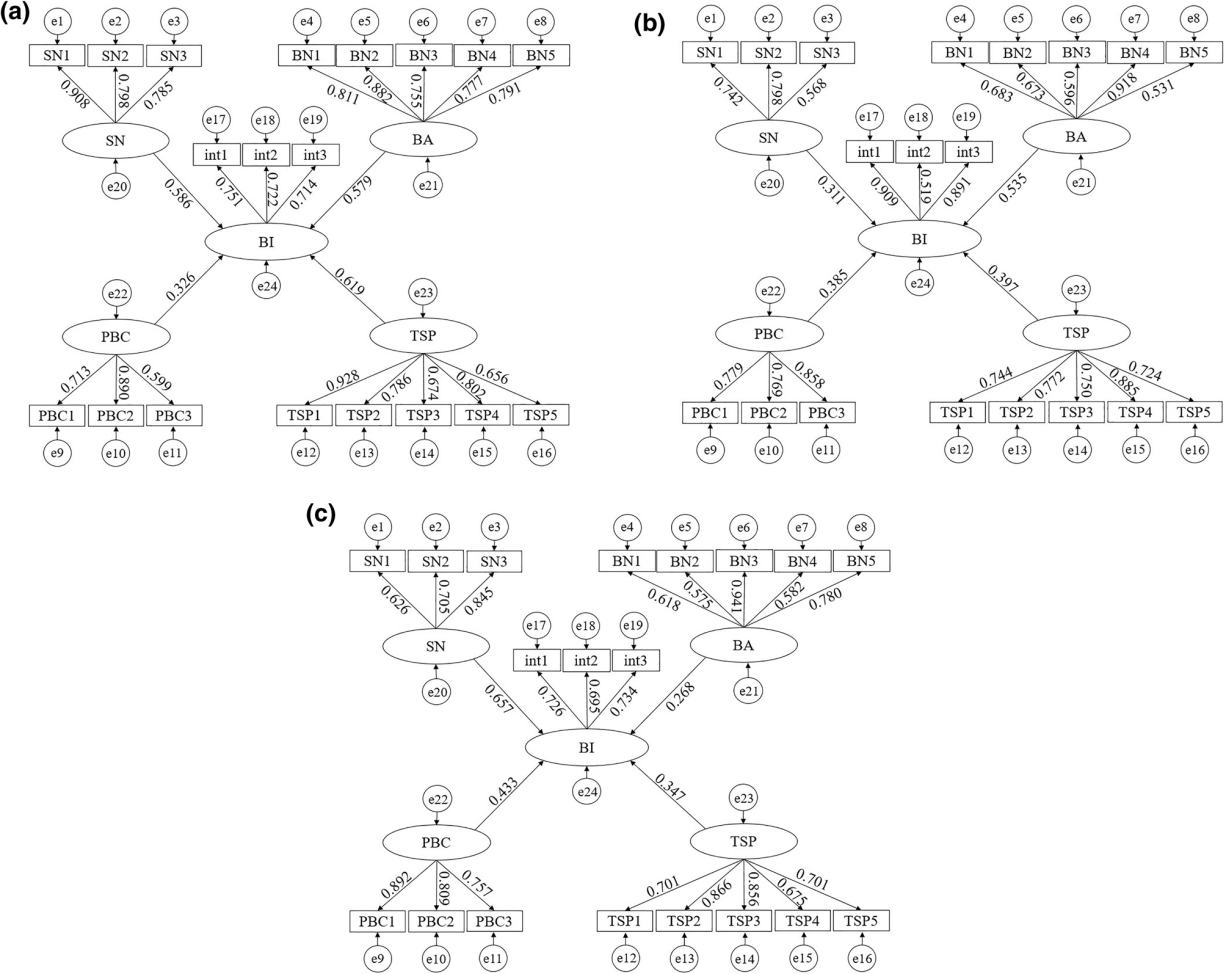
Structural equation model. (**a**–**c**) are structural equation models of the behavioral intention of subway, taxi, and bus travel choice, respectively.

The SEM of the three mode choice, namely, subway, taxi, and bus, are established respectively (Fig. 3).

AMOS 26.0 was used to estimate the initial parameters for SEM. Chi-square degrees of freedom (CMIN/DF), approximate root mean square error (RMSEA), goodness of fit index (GFI), comparative fit index (CFI), normed fit index (NFI), Tucker-Lewis index (TLI), and incremental fit index (IFI) were used to assess the fitness of model. The result meeting the standard requirements and suitable for subsequent analysis (see Table 4)^51^.

**Table 4:** Goodness of fit for structural equation models.

Indicators	CMIN/DF	GFI	CFI	NFI	TLI	IFI	RMSEA	RMR	SRMR
Criteria	1 ~ 5	> 0.8	> 0.8	> 0.8	> 0.8	> 0.8	< 0.08	< 0.05	< 0.05
Subway	1.81	0.927	0.971	0.939	0.959	0.972	0.052	0.021	0.049
Taxi	1.68	0.93	0.984	0.962	0.98	0.984	0.047	0.021	0.028
Bus	1.73	0.937	0.976	0.945	0.968	0.976	0.048	0.025	0.031

Data source: Compiled by the author from AMOS 26.0 software runs.

The path coefficients were normalized^52^, as shown in Table 5. It is evident that each latent variable has a significant impact on travel modal shift, and all the proposed hypotheses: H1 ~ H4 are valid. The standardized path coefficient reflects the magnitude of the direct influence between variables. For subway commuters, TSP (0.293), SN (0.278), BA (0.274), and PBC (0.155) exhibit a decreasing trend in modal shift intention. It can be inferred that subway can significantly avoid traffic issues such as detours, road slope, and congestion on narrow sections caused by one-way streets or river crossing that road traffic may encounter. Other studies have also reached similar conclusions^53^. The influence of BA (0.329), PBC (0.236), TSP (0.244), and SN (0.191) on commuters’ intention to choose taxi decreased progressively. It is clearly linked to the increased emphasis on the service quality for commuters. This finding aligns with the research results^12^. Furthermore, SN (0.385), PBC (0.254), TSP (0.204), and BA (0.157) had a decreasing impact on commuters’ intention of by bus. Bus travel not only benefits from social policies, but as one of the more established modes of public transport within the city, it also offers advantages in terms of economy and convenience. It is almost no obstacles to bus commuters. Chen et al. also illustrates this point^54^.

**Table 5:** Hypothesis test and path estimate.

Hypothesis	Subway			Taxi			Bus		
	Std estimate	Adapt estimate	*p*	Std estimate	Adapt estimate	*p*	Std estimate	Adapt estimate	*p*
H1:TI ← SN	0.586	0.278	***	0.311	0.191	***	0.657	0.385	***
H2:TI ← BA	0.579	0.274	***	0.535	0.329	***	0.268	0.157	***
H3:TI ← PBC	0.326	0.155	***	0.385	0.236	***	0.433	0.254	***
H4:TI ← TSP	0.619	0.293	***	0.397	0.244	***	0.347	0.204	***

***Indicates a significant level of 0.001. Data source: Compiled by the author from AMOS 26.0 software runs.

### Travel modal behavior

We use Stata 16 software to estimate the parameters and analyze the significance level of the model. It is assumed that the four variables of subjective norm, behavioral attitude, perceived behavioral control, and terrain spatial perception of commuters in valley cities are random variables that follow a normal distribution. This is because the RPLM with random coefficients is non-closed and cannot be solved directly, so it can only be solved using the imitation method^55^. In random sampling, the Halton sequence has a better sampling effect^56^. Therefore, this paper utilizes the Monte Carlo simulation method to estimate the maximum likelihood parameters and employs the Halton sampling method to extract 500 samples. The final results are shown in Table 6.

**Table 6:** Parameter estimation results of random parameter Logit model.

Choice	Variable	Odd ratio	Coefficient	Std. err	*p*
σ/Normal	sd(Subjective norms)	0.307	0.307	0.231	> 0.100
	sd(Behavior attitude)	0.263	0.263	0.222	> 0.100
	sd(Perceived behavior control )	0.424	0.424*	0.236	< 0.100
	sd(Terrain spatial perception)	0.615	0.615***	0.237	< 0.010
μ	Subjective norms	1.197	0.180**	0.071	0.011
	Behavior attitude	1.243	0.217***	0.081	0.007
	Perceived behavior control	1.162	0.150**	0.075	0.047
	Terrain spatial perception	1.195	0.179**	0.077	0.020
Subway	(base alternative)				
Taxi	con_	53.365	3.977***	1.413	0.005
	Gender	2.874	1.056***	− 0.314	0.001
	Age	1.192	0.176	− 0.168	0.294
	Monthly income	2.917	1.071***	− 0.202	0.000
	Private car ownership	1.650	0.501	0.306	0.102
	Length of residence/year	0.454	− 0.789**	0.321	0.014
	Workplace-residence distance/km	0.347	− 1.057***	0.314	0.001
	Road slope between OD/%	0.773	− 0.258**	0.122	0.034
	Grouping characteristic between OD	0.396	− 0.928***	0.310	0.003
	Congestion between OD	0.325	− 1.125***	0.306	0.000
	River-crossing demand between OD	0.595	− 0.520*	0.280	0.064
Bus	con_	1696.955	7.437***	1.644	0.000
	Gender	0.850	− 0.163	0.263	0.536
	Age	1.663	0.509***	0.163	0.002
	Monthly income	0.804	− 0.218	0.154	0.157
	Private car ownership	0.408	− 0.896***	0.327	0.006
	Length of residence/year	0.501	− 0.690**	0.318	0.030
	Workplace-residence distance/km	0.488	− 0.718**	0.295	0.015
	Road slope between OD/%	0.755	− 0.281**	0.114	0.013
	Grouping characteristic between OD	0.510	− 0.674**	0.288	0.019
	Congestion between OD	0.393	− 0.934***	0.291	0.001
	River-crossing demand between OD	0.520	− 0.654**	0.278	0.019

***p < 0.01, **p < 0.05, *p < 0.1. Data source: Compiled by the author from Stata 26.0 software runs. The results calculated by the Stata official command only provide the Z and P values of the random parameter mean, and do not provide the significance statistics of its standard deviation. The author calculates the Z statistic by the formula (Z statistic = estimated value/standard error), and compares it with the critical value table to obtain the significance of the standard deviation.

It can be seen from the results in Table 6 that the standard deviations of SN and BA are not significant, so we have assumed their coefficients as fixed parameters for re-modeling, and the results are shown in Table 7.

**Table 7:** Parameter estimation results of random parameter Logit model.

Choice	Variable	Odd ratio	Coefficient	Std.err	*p*
σ/Normal	sd(Perceived behavior control )	0.603	0.603*	0.362	< 0.100
	sd(Terrain spatial perception)	0.612	0.612***	0.192	< 0.010
μ	Perceived behavior control	0.144	0.144**	0.064	0.024
	Terrain spatial perception	0.163	0.163**	0.068	0.016
Subway	(base alternative)				
Taxi	con_	13.206	2.581	1.750	0.140
	Gender	2.833	1.041***	0.268	0.000
	Age	1.163	0.151	0.154	0.327
	Monthly income	2.763	1.016***	0.166	0.000
	Private car ownership	1.682	0.520*	0.271	0.055
	Length of residence/year	0.510	− 0.672**	0.280	0.016
	Work-residence distance/km	0.379	− 0.970***	0.283	0.001
	Road slope between OD/%	0.808	− 0.213**	0.108	0.048
	Grouping characteristic between OD	0.428	− 0.849***	0.273	0.002
	Congestion between OD	0.368	− 0.999***	0.265	0.000
	River-crossing demand between OD	0.641	− 0.445*	0.256	0.082
	Subjective norms	0.681	− 0.384*	0.205	0.061
	Behavior attitude	1.542	0.432**	0.208	0.038
Bus	con_	14,062.330	9.551***	1.694	0.000
	Gender	0.940	− 0.062	0.226	0.783
	Age	1.496	0.403***	0.131	0.002
	Monthly income	0.850	− 0.163	0.125	0.191
	Private car ownership	0.504	− 0.685***	0.246	0.005
	Length of residence/year	0.587	− 0.532**	0.255	0.037
	Work-residence distance/km	0.528	− 0.639**	0.250	0.011
	Road slope between OD/%	0.807	− 0.215**	0.093	0.021
	Grouping characteristic between OD	0.610	− 0.494**	0.244	0.043
	Congestion between OD	0.440	− 0.820***	0.240	0.001
	River-crossing demand between OD	0.564	− 0.572**	0.233	0.014
	Subjective norms	0.786	− 0.241	0.179	0.178
	Behavior attitude	0.523	− 0.649***	0.196	0.001

***p < 0.01, **p < 0.05, *p < 0.1. Data source: Compiled by the author from Stata 26.0 software runs.

### In-sample fit and predictions

In order to verify the rationality and effectiveness of the SEM-RPLM, we constructed traditional Multinomial Logit (MNL) model and SEM-MNL model as comparison models. Table 8 presents the fit indices for the three models. Firstly, all the models can effectively explain the data. Secondly, the SEM-RPLM outperforms the other two models in all performance indicators^57^.

**Table 8:** Models fitting index.

Model	LL (β)	LL (0)	Pseudo R^2^	AIC	BIC
MNL	− 2072.680	− 2413.729	0.141	4155.359	4233.505
SEM-MNL	− 2068.796		0.143	4151.592	4260.996
SEM-RPLM	− 661.697		0.722	1383.395	1521.917

Data source: LL (0), LL (β), and Pseudo R^2^ obtained by the authors from the results of SPSS 26.0 software runs. AIC and BIC obtained by the authors’ calculations based on formulae.

## Discussion

Our study yields some intriguing conclusions as following.

### Individual socioeconomic attributes

The primary demographic groups of subway users include women, middle-aged and young adults, local individuals, those with middle to low income, car-free individuals. Compared to men, women are more hesitant to travel by car, possibly due to the higher physical demands of driving in the complex urban terrain of the river valley type city^58^. Older commuters tend to prefer traveling by car and bus rather than by subway. After consulting the research of Talbot et al., we attempt to explain the possible reason for this phenomenon. It may be attributed to the complexity of subway rides and the fact that accessibility is not as good as the other two options^59^. As a result, older travelers in the river valley-type city who opt for subway may encounter poor terrain conditions when walking away from the station. Similar to the findings of plain cities commuting behavior studies, individuals with higher income and more private cars are more likely to opt for taxis. This indicates that commuters with higher income and more private cars are not easily persuaded to commute by subway due to the relatively low terrain spatial perception of river valley-type cities. Local commuters prefer commuting by subway. The reason may be that local residents are more likely to have private cars and know more about the complex terrain space of the valley-type city^60^.

### Objective built environment attributes

Our study reveals that commuters in river valley-type cities are more likely to commute by subway if they have to detour between OD to cross the river in the river valley-type cities via a bridge.

When commuters travel during peak times, they tend to choose the subway, a conclusion that can also be found in the research findings of Luan et al^61^. The advantages of the subway, such as being unaffected by longer traffic congestion in the river valley-type city than in plain cities wasn’t considered by them, but we were. The advantages of subway and taxi are more prominent in long-distance commute, especially in river valley-type cities with higher average workplace-residence separation. With the increase in commuting distance, the subway becomes more cost-effective compared to using a taxi, and its appeal to passenger flow becomes more evident. Individuals who commute between urban groups will be more likely to use the subway. This is because traffic development is uneven of the group center and the outskirts in the river valley-type city. Commuting between urban groups often involves longer distances compared with commuting within the urban group, making the subway a more convenient option compared to road traffic. As we had hypothesized, our study found that a steep road slope effectively promotes the use of the subway^4^. There is no doubt that the subway offers a smoother ride in the carriage compared to other road public transportation.

### Psychological perception attribute

SN has a significant impact on travel choices, which is consistent with previous studies^62^. On the one hand, the road resources in river valley-type cities are limited, the single passenger flow direction very conducive to the development of subways with agglomeration effects. On the other hand, the subway in river valley-type cities is not affected by road traffic issues like river crossings, detours, slopes, intergroup travel, or peak congestion. Therefore, the subway is favored by more residents, friends, and society. The subway clearly serves multiple purposes, including cost-effectiveness, speed, environmental protection, and the advantages of the above compared to road traffic. Therefore, commuters have the most positive BA to the subway. When it comes to PBC, considering the long average commute distance in the river valley-type city, commuting by road traffic is likely to experience increased risk of congestion and lateness. Additionally, the presence of slopes and complex road conditions may contribute to an uncomfortable ride experience for commuters. Subway travel imposes no burden on commuters in terms of travel time, physical exertion, cost, or carbon emissions. It is worth mentioning that this paper introduces a new indicator, TSP, based on the TPB. Our research shows that, as commuters’ TSP increases, the likelihood of commuting by subway also increases. It is evident that subway does not have to take detours when encountering one-way street sections or rivers in the region, unlike other road traffic modes. Additionally, it is not affected by road slope. Moreover, in the long-distance commute, subway can reach the destination faster and is not affected by road traffic congestion in the bee-waist sections in river valley-type cities.

Among the four psychological perception variables, PBC ~ N (0.150, 0.424^2^) and TSP ~ N (0.179, 0.615^2^). The mean values of the parameters of PBC and TSP are significant and positive. This implies that in river valley-type cities, the higher PBC and TSP of commuters for a specific mode of public transportation, the more likely commuters are to choose this mode. The absolute value of the mean parameter of TSP is larger, indicating that TSP is a more important factor affecting commuters in river valley-type cities. The significant standard deviation values of both parameters suggest heterogeneity of PBC and TSP among commuters in river valley-type cities. Among them, the absolute value of the standard deviation of TSP is larger, indicating that commuters in river valley-type cities have greater differences in TSP^55,63^. We suspect that the potential source of heterogeneity in individual perceptions is the variation in individual socio-economic attributes. For example, a younger commuter may perceive the slope of a road differently than an older commuter^15^. Further analysis is needed to explore the moderating effect to determine this hypothesis. This part of the study is currently underway.

## Conclusions

### Contributions

This research contributes to literature in the following three important ways. First, we study the travel mode choice of commuters in the river valley-type city. On the basis of previous studies, we extend the influencing factors according to the characteristics of the river valley-type city, and introduce TSP to expand TPB. The five observation variables corresponding to the TSP proposed by the authors can well explain the latent variable. Secondly, under the theoretical framework of the extended TPB, the SEM-RPLM is established to reveal the significant factors influencing commuters’ behavior and intention of travel mode choice in the river valley-type city. Compared to the MNL model and the SEM-MNL model, the SEM in SEM-RPLM accurately quantifies the potential psychological perception, and the RPLM considers the heterogeneity of commuters’ sensitivity to influence factors. This makes the model superior in terms of explanatory power and prediction accuracy. Finally, we have proposed feasible solutions to address the traffic issues in the river valley-type city.

### Suggestions

Metro operators must prioritize minimizing the gap with taxi in terms of convenience and accessibility while maintaining safety and cost-effective advantages. For instance, implementing measures like adding parallel lines on both sides of the river in the region and designing parallel branches resembling fish bones in the urban group center can provide convenience for commuters who need to cross the river in river valley-type cities. Accelerate the construction of mixed-use land within the group to promote a balance between workplace and residence, and increase the proportion of intergroup commute where subway has advantages. Reasonable planning of the layout of subway stations in challenging terrain environments and improving walking tracks, overpasses, underpasses, and other facilities near the stations can help reduce obstacles such as walking uphill or taking detours before and after using subway, thus encouraging the use of subway. Add subway exits at the stations located at the centers and the junctions of urban group to alleviate congestion in densely populated areas. The transportation authorities should utilize various methods to extensively promote the benefits of subway travel over road transportation in river valley-type cities, particularly targeting the non-local population with limited familiarity with the terrain of river valley-type cities. Government policies aimed at promoting low-carbon mobility can also reinforce the SN that influence commuters’ decisions to choose subway. By publicizing the advantages of subway over road traffic in river valley-type cities, such as reduced impact from the river, road slopes, detours, and peak congestion at urban group centers and junctions in the region, as well as more convenient travel between urban group, it can enhance commuters’ attitudes towards subway commuting, perceived behavior control, and terrain space perception. This can also attract commuters who typically use road traffic to opt for subway commuting.

### Limitations

The study also has some limitations. Firstly, previous studies have seldom examined public transportation commuting behavior in river valley-type cities from the perspective of terrain spatial perception. Therefore, the observed variables corresponding to the latent variables of terrain spatial perception lack the reference of a mature scale. Secondly, this paper focuses solely on studying the public transportation commuting behavior in river valley-type cities. A comparison of public transportation commuting behavior with that of plain cities of the same scale is currently under further investigation. Thirdly, the results of this study indicate that there is heterogeneity in the TSP of commuters in river valley-type cities. However, further research is needed to explore the moderating effects.

## Data Availability

All data generated or analyzed during this study are included in this published article.
